# Decoding lower-limb movement attempts from electro-encephalographic signals in spinal cord injury patients

**DOI:** 10.1063/5.0297307

**Published:** 2026-01-20

**Authors:** Laura Toni, Valeria De Seta, Luigi Albano, Daniele Emedoli, Aiden Xu, Vincent Mendez, Filippo Agnesi, Sandro Iannaccone, Pietro Mortini, Silvestro Micera, Simone Romeni

**Affiliations:** 1 Modular Implantable Neurotechnologies (MINE) Laboratory, Università Vita Salute San Raffaele & Scuola Superiore Sant'Anna, 20132 Milano, Italy; 2 The BioRobotics Institute, Health Science Interdisciplinary Research Center, and Department of Excellence Robotics and AI, Scuola Superiore Sant'Anna, 56127 Pisa, Italy; 3 Bertarelli Foundation Chair in Translational Neuroengineering, Neuro-X Institute, Ecole Polytechnique Federale de Lausanne (EPFL), 1015 Lausanne, Switzerland; 4 CHUV, Department of Clinical Neurosciences, University Hospital Lausanne, Lausanne, Switzerland; 5 Neurosurgery and Gamma Knife Radiosurgery Unit, IRCCS Ospedale San Raffaele, 20132 Milan, Italy; 6 Department of Rehabilitation and Functional Recovery, IRCCS Ospedale San Raffaele, 20132 Milan, Italy

## Abstract

Restoring lower-limb function in patients with severe spinal cord injury (SCI) remains challenging. Spinal cord stimulation may enhance and reinstate lower-limb movements, but it is either used in open-loop control or its control depends upon residual motor functions, limiting its applicability in severely paralyzed individuals. The decoding of motor intentions from cortical signals may provide an interesting alternative in such cases. Electroencephalography (EEG) is an ideal solution since it is noninvasive and has been employed diffusely in the past to decode upper-limb movement intentions. Nonetheless, its application in lower-limb control remains underexplored. In this study, we investigated whether EEG can be used to decode lower-limb movement correlates in four SCI patients with varying injury severity during attempted left/right hip flexion or knee extension across four experimental sessions. We performed statistical analysis of event-related desynchronization/synchronization and machine learning classification to evaluate single and multi-window decoding performance. Our results suggest that EEG signals can often differentiate lower-limb movement attempts from rest, whereas decoding of left vs right and hip vs knee movements was more elusive. Left vs right decoding accuracy was improved through multi-window decoding, showing multiple sessions with above-chance results. In one patient, it was possible to attain above-chance three-class decoding (left/right/rest). Discriminating hip and knee movements proved more challenging. These findings establish a baseline for EEG decoding of lower-limb motor attempts in severely paralyzed individuals and pave the way for the development of brain-controlled neuroprosthetic systems.

## INTRODUCTION

Spinal cord injuries (SCIs) have severe consequences on the quality of life of the affected patients^1–5^ and constitute a relevant societal problem, with over 15 × 10^6^ cases worldwide (source: World Health Organization), and annual healthcare costs estimated at more than one million dollars per patient throughout their life.^6^ The loss of lower-limb motor functions is an important consequence of SCI, reducing patients' mobility and independence, contributing to place lower-limb function restoration among the priorities of such individuals.^4^ Activity-based physical therapy has shown promise in the rehabilitation of patients maintaining residual motor functions after the SCI (also referred to as incomplete SCI patients).^7–12^ However, evidence that intensive rehabilitation practice may lead to motor functional improvements in complete SCI patients remains limited.^13^

Electrical spinal cord stimulation (SCS) has shown the potential to improve or reinstate functional movements in both incomplete^14–18^ and complete^19–21^ SCI patients. Even though the final goal of SCS interventions is to assist patients in their everyday life, providing targeted stimulation protocols that address their specific functional needs,^21^ spatiotemporal control of stimulation in unstructured settings remains challenging. The possibility to employ kinematic or myoelectric control strategies for electrical stimulation has been shown in the past^22–25^ but requires residual motor functions in the target patients, making control approaches for complete SCI patients particularly challenging. The best candidate to control stimulation in these cases would be the development of a “brain–spine interface,” directly linking cortical motor commands to SCS.^21,26–29^ A first in-human proof-of-concept of an invasive brain–spine interface has recently been presented using electrocorticography (ECoG).^30^ Despite the promising results, ECoG requires a surgery involving the exposure of part of the brain, leading to the risk of infections,^31^ thus limiting its adoption. In addition, the procedure appears overly invasive, given that walking is not the first concern for individuals with SCS, who typically prioritize bowel, urinary, and sexual functions.^4^

Electroencephalography (EEG) has been proposed as an interesting noninvasive alternative to ECoG for brain–spine interfaces.^32,33^ EEG has a lower signal-to-noise ratio^34^ and spatial resolution^35^ than ECoG, but several studies have shown the feasibility of decoding upper-limb motor attempts^36–40^ and movement intentions^41^ using EEG in individuals with cervical SCI. Movement-related cortical modulations are normally studied in the frequency domain across theta (4–8 Hz), alpha (8–12 Hz), beta (12–30 Hz), and gamma (30–100 Hz) frequency bands. Event-related desynchronization/synchronization (ERD/ERS),^42^ quantifying the change in signal band-power between a task and a rest intervals, are important tools employed in EEG decoding and EEG-based brain–computer interfaces (BCIs). In general, movement execution and motor imagery have been associated with low frequency (theta, alpha, and beta band) ERD and high frequency (gamma band) ERS.^43–46^

EEG decoding of lower-limb movements remains less explored compared to upper-limb decoding, and it is generally accepted to be more challenging due to the anatomical location of the lower-limb cortical areas, located in a small region within the central sulcus. In healthy subjects, EEG studies on lower-limb decoding, often coupled with functional electrical stimulation^47^ or exoskeletons,^48,49^ have shown the possibility to discriminate task vs rest trials,^50^ distinguish different complex movements one from the other,^50^ and generally achieve above chance left vs right foot motor imagery and execution.^51,52^ Recent work has also expanded to include decoding of movement onset,^53,54^ continuous estimation of kinematic variables,^55–57^ and classification of motor imagery patterns.^58,59^ When moving to paralyzed patients, only a small subset of paralyzed individuals exhibited statistically significant beta band ERD or other detectable correlates of motor attempt.^44^ Nevertheless, studies testing on patients remain limited, particularly those involving motor-complete injuries.^32,60^ Gaining a clearer understanding of how much lower-limb activity can be decoded from noninvasive EEG in SCI patients is key to improving neuroprosthetic control and the development of practical noninvasive brain–machine interfaces.

In the present work, we investigate the feasibility of decoding lower-limb movement attempts from EEG signals in individuals with severe SCI. We collected EEG data from four SCI patients (one motor incomplete SCI patient suffering from distal denervation and three motor-complete SCI patients) during the attempted execution of four movements essential for walking (left/right hip flexion and knee extension) across four recording sessions. We characterize spectral differences between task and rest intervals and provide a statistical characterization of their discriminability. Then, we use machine learning to classify attempted movements vs rest and motor preparation vs rest and explore the discriminability of different movement types (left vs right and hip vs knee) using spectral features. This work aims to providing a thorough baseline for these decoding tasks in severely paralyzed patients, in order to evaluate the feasibility of EEG-controlled electrical stimulation protocols and to guide their development considering which decoding tasks are more reliable on this patient cohort.

## RESULTS

**FIG. 1.**
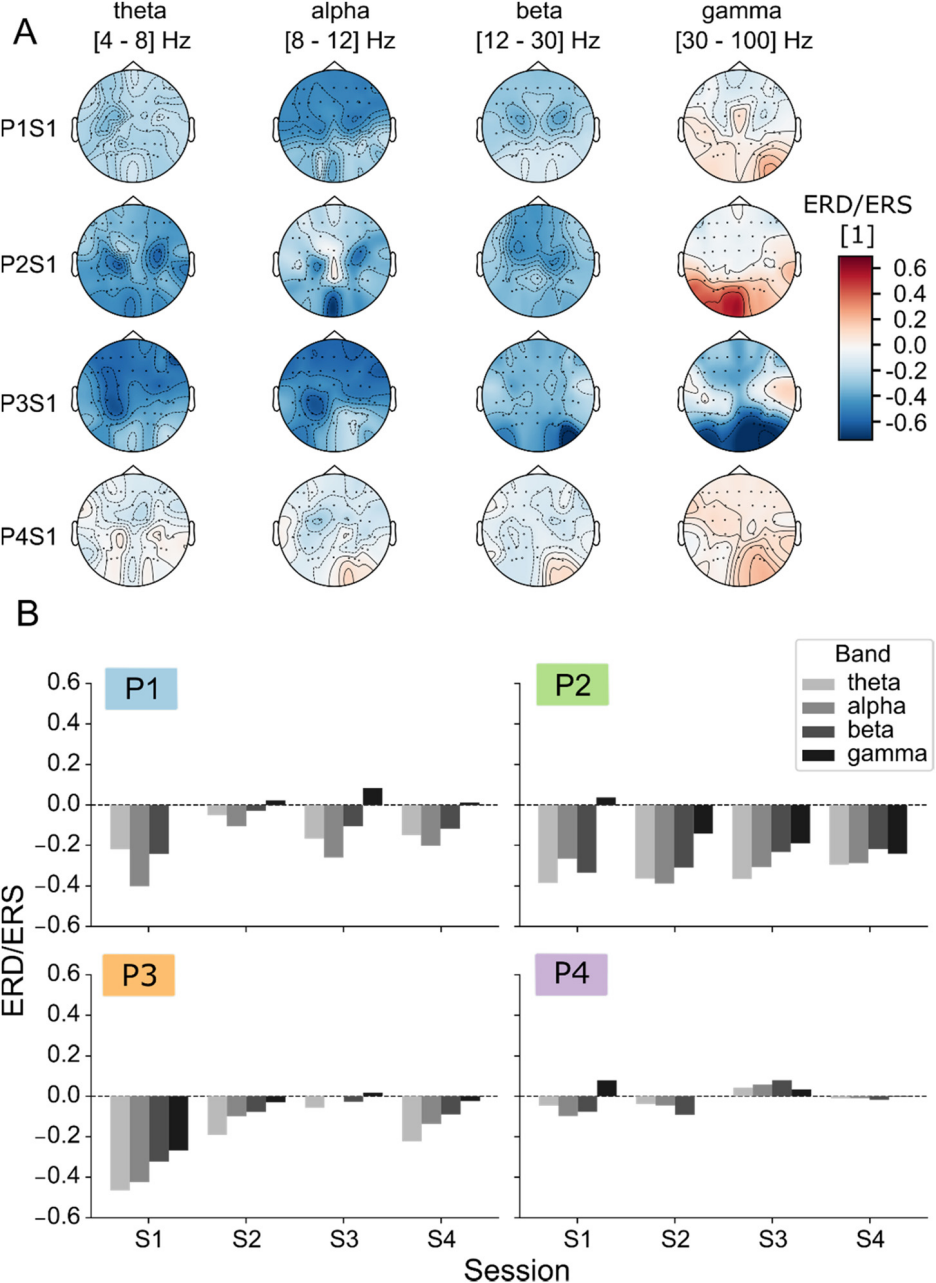
ERD/ERS topographies. (a) Event-related desynchronization/synchronization (ERD/ERS) topographies in theta (4–8 Hz), alpha (8–12 Hz), beta (12–30 Hz), and gamma (30–100 Hz) bands for the first session across all patients, computed as the ratio of the PSD between the task (4–7 s) and rest (8–11 s) phases. In the topographical maps, the standard position of the channels with respect to the subject head is indicated through solid dots (supplementary material Fig. 2). The color indicates the ERD/ERS interpolated from channel-wise values to the whole scalp. Solid and dashed lines indicate, respectively, positive- and negative-value level lines. (b) Average ERD/ERS for all subjects and sessions, obtained by averaging among all channels. Different colors indicate that the values refer to a specific frequency band. P: patient, S: session.

When inspecting the band-wise ERD/ERS for the motor attempt vs rest periods across the sensor topography, we can see that ERD is generally dominating in the theta, alpha, and beta bands [see Fig. 1(a) for the first session from each patient, and supplementary material Figs. 3 and 4 for all other sessions]. While ERDs/ERSs are generally diffused across large parts of the scalp, in some cases, focal ERDs can be spotted in the central channels (see, for example, beta band in P1S1; theta, alpha, and beta bands in P2S1; theta and alpha bands in P3S1). When inspecting the average ERD/ERS per band [Fig. 1(b)], we can notice that lower frequencies tend to present larger ERDs. In the gamma band, ERSs can be seen sporadically. We observe that, except for a marked tendency toward task-related desynchronization, it is very difficult to establish other repeatable trends.

**FIG. 2.**
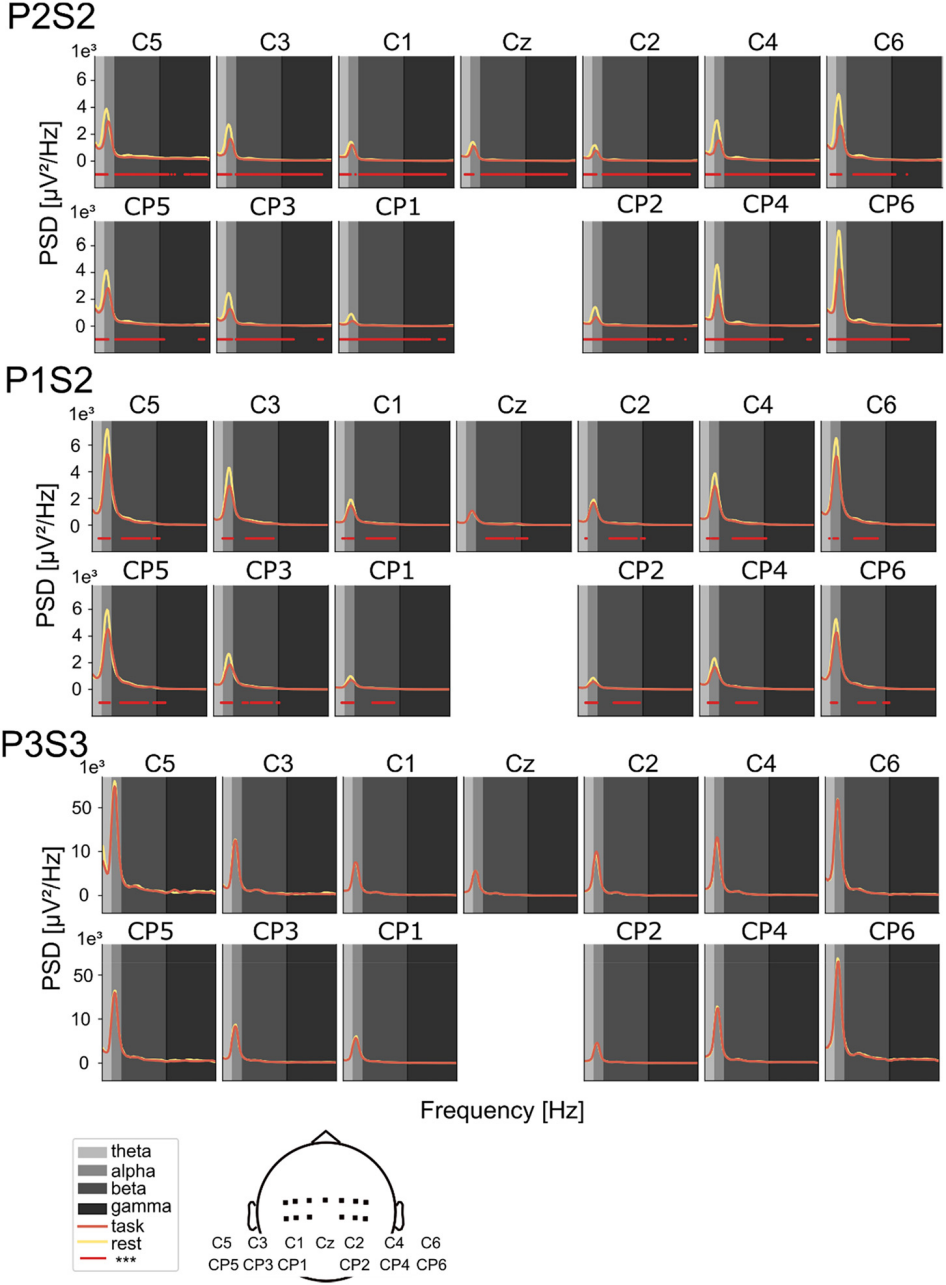
Spectral power differences between movement and rest phases in central channels. Power spectral density (PSD) comparisons are shown for three representative sessions: a high-performing session (P2S2), a low-performing session (P3S3), and a typical session (P1S2). PSDs were computed using the multitaper method across the 4–100 Hz range, focusing on 13 central EEG channels (represented on the bottom) associated with sensorimotor processing. Significant differences between movement (task) and rest periods were identified using a non-parametric cluster-based permutation test (p < 0.05), with spatial clustering based on channel adjacency. Frequencies that have significant differences are underlined in red.

A more thorough characterization of spectral differences between motor attempt vs rest intervals is presented in Fig. 2, with significant space-frequency clusters shown across the central channels. We can see the characteristic 1/f behavior of the spectrum typical of cortical signals, where a peak is generally present in correspondence with alpha/beta frequencies. Statistical differences generally correspond to low power frequency bands (theta, alpha, beta, but very rarely gamma), including but not limited to frequency intervals exhibiting the above-mentioned spectral peaks. Supplementary material Fig. 5 shows results for all subjects and sessions. Patients P1-2 present statistically significant clusters in theta, alpha, and beta bands across all sessions, while patients P3-4 show significant clusters only for the first session.

**FIG. 3.**
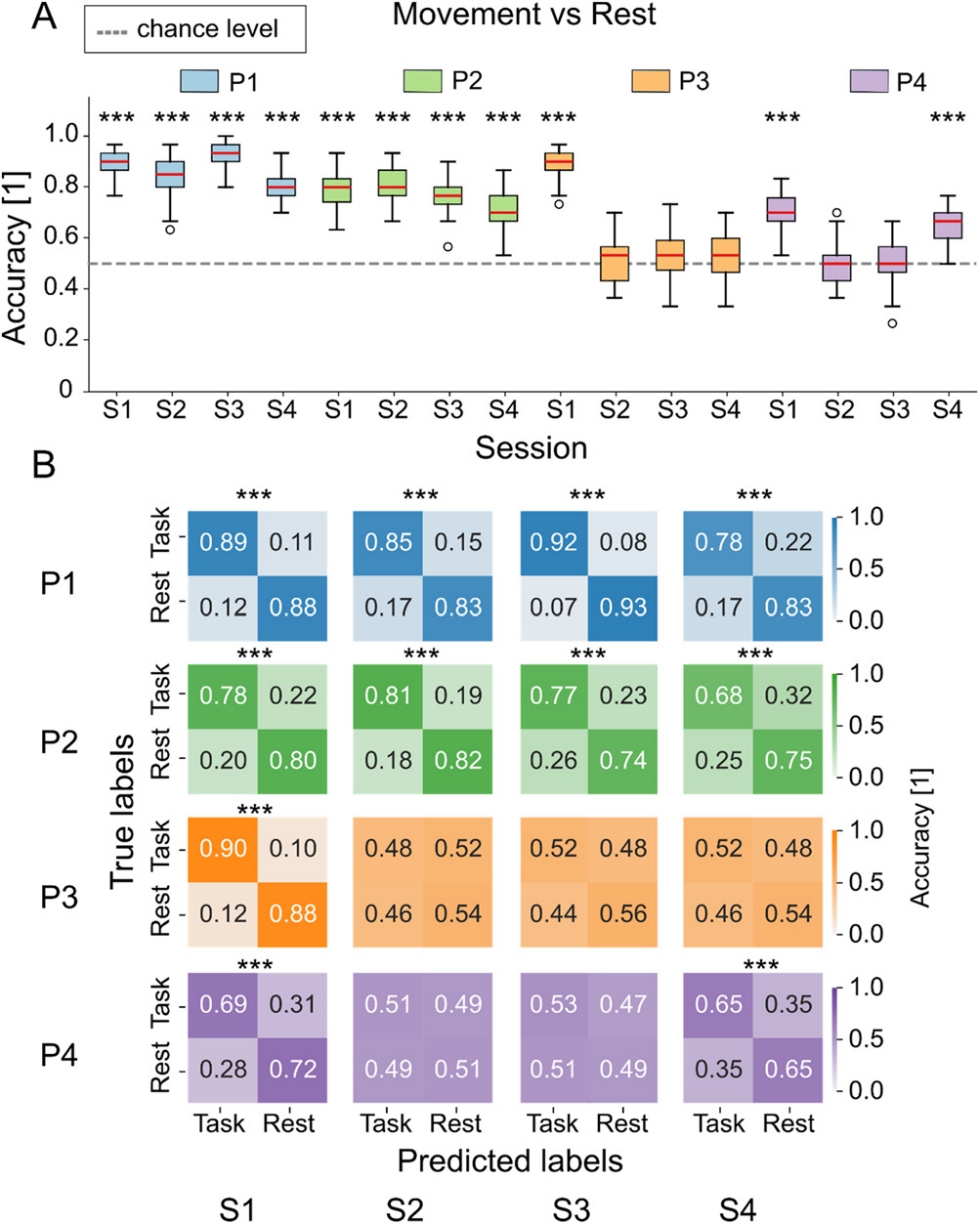
Movement vs rest classification across sessions. Statistically significant sessions are marked with (*). (a) Boxplots of classification accuracy computed over 50 resampling iterations. Red lines indicate the median, whiskers represent the interquartile range (25th–75th percentile), and empty circles denote outliers. (b) Confusion matrices averaged over 50 resamples and normalized by the total number of true labels per class. *p* < 0.05/N (^*^), *p* < 0.01/*N* (^**^), and *p* < 0.001/*N* (^***^), with *N* = 16 (number of sessions × number of patients). P: patient, S: session.

In Fig. 3, we show the results of single-trial movement attempt vs rest decoding both in terms of classification accuracies across all Monte Carlo resamples [Fig. 3(a)] and average confusion matrices [Fig. 3(b)]. We can see that patients P1-2 obtain, in general, above-chance results, while patients P3-4 show much lower performances, with several sessions not reaching significance in the task vs rest classification. In supplementary material Fig. 6, we can see the performance of the movement preparation vs rest classification, leading as expected to lower performances than the movement attempt vs rest one, but reaching above-chance decoding in most sessions where movement attempt vs rest was significantly above chance. In supplementary material Fig. 7, we can see that the performance of the decoding of single tasks (hip, knee, right, and left) vs rest does not vary substantially for a given patient and session when we want to discriminate, e.g., hip vs rest or knee vs rest. Finally, supplementary material Fig. 11 shows that using all channels for classification yields performance comparable to that obtained using only central channels, whereas applying a common spatial patterns (CSP) decomposition generally results in lower accuracy, particularly when only five components are used.

**FIG. 4.**
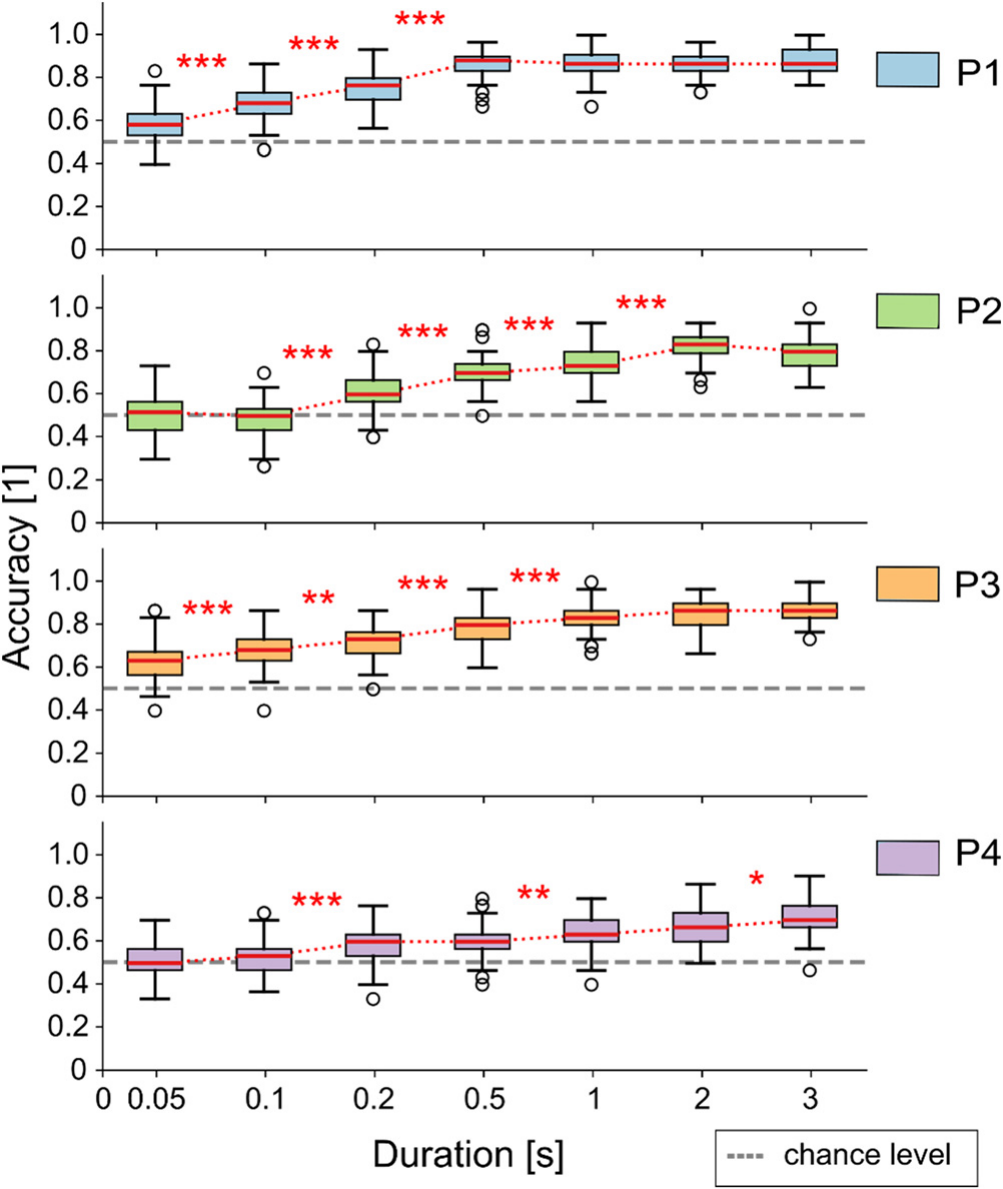
Effect of time window length on movement vs rest classification in Session 1. Boxplots show classification accuracy across different window durations for Session 1 of each patient, computed over 50 resamples. Red lines indicate the median, whiskers represent the 25th–75th percentiles, and empty circles denote outliers. *p* < 0.05/*N* (^*^), *p* < 0.01/*N* (^**^), and *p <* 0.001/*N* (^***^), with N = 7. P: patient.

The variation of task vs rest classification accuracy, when decoding signal windows with increasing duration, is shown in Fig. 4. We can see that performances tend to increase with window duration, reaching in most cases a plateau. Such plateau shows some degree of inverse correlation with the average performance in task vs rest classification, so, for example, it occurs for the lowest duration value (0.5 s) in patient P1 (exhibiting the highest decoding performance) and for the highest duration value (3 s) for patient P4 (exhibiting the lowest decoding performance), while it has intermediate values for patients P2 and P3 (2 and 1 s, respectively).

**FIG. 5.**
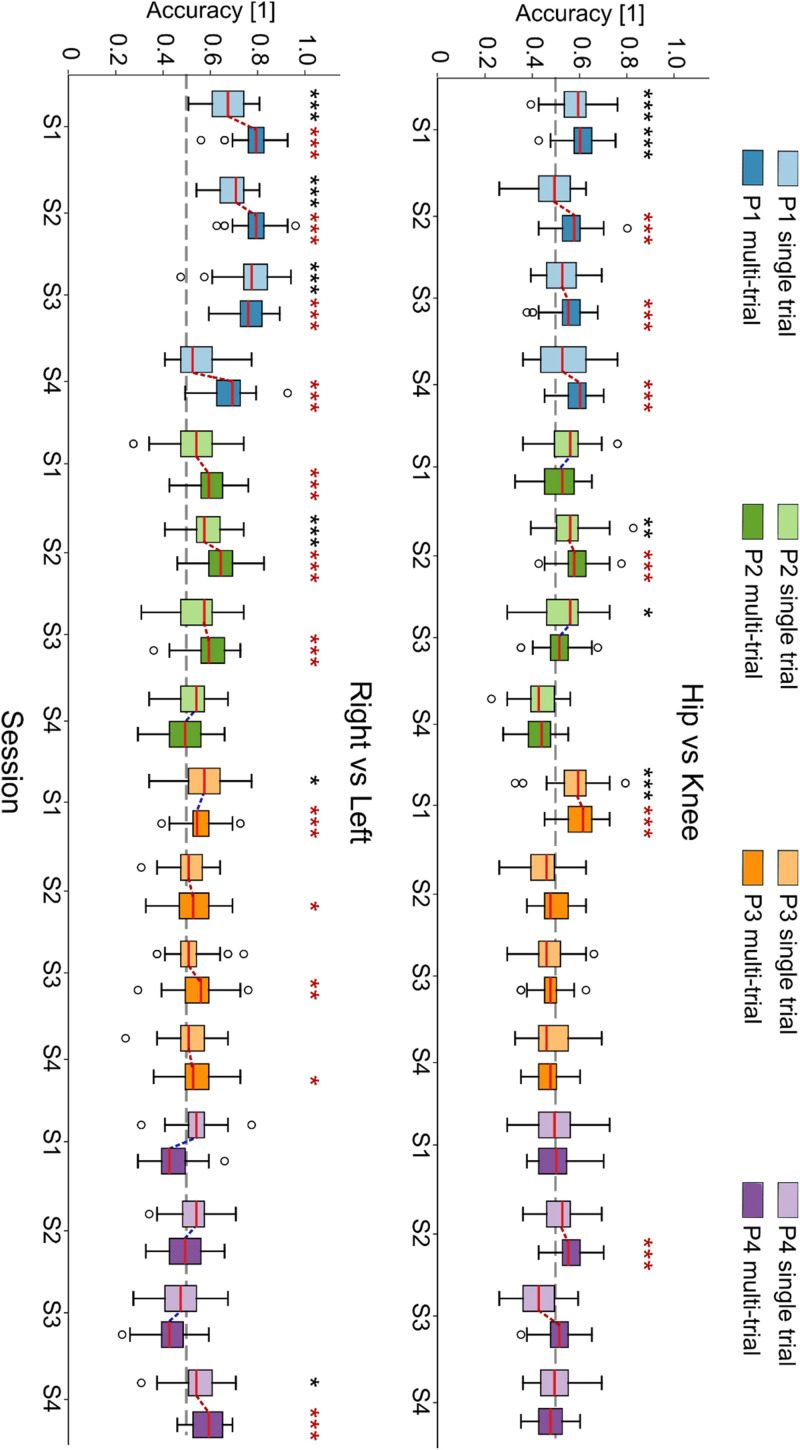
Classification performance across movement types. Boxplots showing classification accuracy for (a) right vs left movement discrimination and for (b) hip vs knee movement discrimination, based on a single-trial and a multi-window approach. Boxplots are computed over 50 resampling iterations, red lines denote the median, and whiskers represent the interquartile range (25th–75th percentile). Statistically significant sessions compared to random classifiers are indicated with black (*) for single-trial classification and with red (*) for multi-window. Red/blue dotted lines connect multi-window sessions that show higher/lower median accuracy compared to single trial. *p* < 0.05/*N* (^*^), *p* < 0.01/*N* (^**^), and *p* < 0.001/*N* (^***^), with *N* = 16 (number of sessions). P: patient.

Figure 5 shows the decoding performance for right vs left [Fig. 5(a)] and knee vs hip [Fig. 5(b)] classification tasks as boxplots of the accuracy distributions, for each patient and session, for both single and multi-window approaches. In general, in single-trial decoding, patient P1 is the only one showing above-chance results across more than one session, and it is the only one where we have found it possible to discriminate hip vs knee attempted movements. We remark in any case that even when statistically above chance, the average decoding accuracy for patients P2-4 in the left vs right task as well as for patient P1 in the hip vs knee task is always below 0.60.

In multi-window decoding, the number of statistically significant sessions approximately doubles for both hip vs knee and right vs left classifications. In such cases, median accuracy over the 50 resamples grows by combining multiple scores together, indicating that information to discriminate the two conditions is actually present. Only in a few sessions in which single-trial accuracy was close to chance level (e.g., knee vs hip: P2S1, P2S3; right vs left: P2S4, P4S1), the median multi-window accuracy decreased. Supplementary material Figs. 8 and 9 present confusion matrices for both single and multi-window classification in statistically significant sessions. In multi-window condition, confusion matrices tend to be diagonal, indicating that the increased accuracy reflects actual classification improvements rather than bias or class imbalance.

**FIG. 6.**
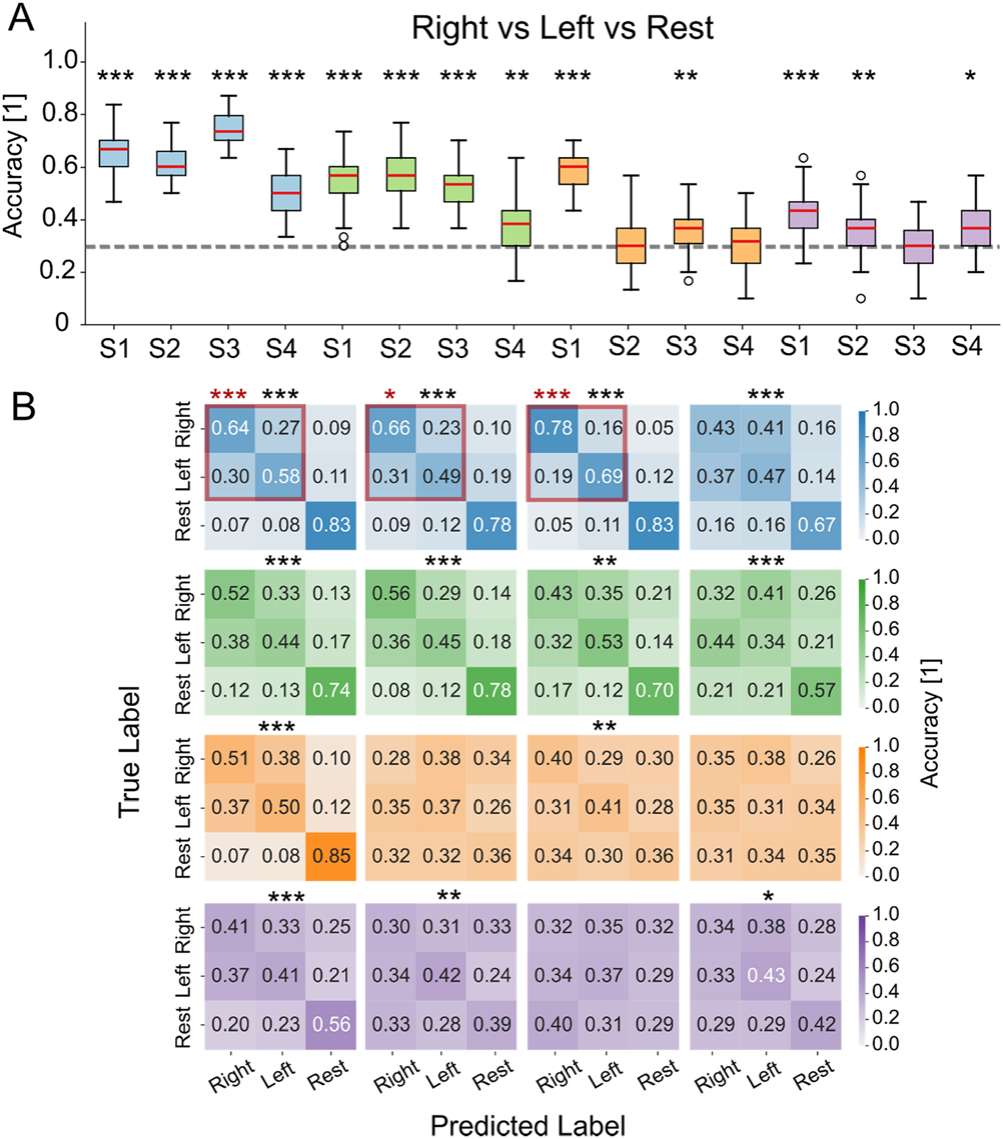
Multi-class classification: right vs left vs rest. Classification performance across all sessions and patients. Statistically significant sessions are marked with (*). Sessions also significant in right vs left classification are additionally highlighted with a red square and red (*). (a) Boxplots show classification accuracy over 50 resamples (median: red line, whiskers: 25th–75th percentile, outliers: empty circles). (b) Confusion matrices averaged over 50 resamples, normalized by true class counts. *p* < 0.05/*N* (^*^), *p* < 0.01/*N* (^**^), and *p* < 0.001/*N* (^***^), with *N* = 16 (number of sessions). P: patient, S: subject.

Finally, we display the results of right vs left vs rest decoding using a three-class classifier in Fig. 6. In Fig. 6(a), boxplots of decoding accuracies across resamplings are shown, whereas in Fig. 6(b), we show the confusion matrices of the classifications. Even though we can see that the three-class accuracy is almost systematically above chance [Fig. 6(a)], this derives mostly by the high capacity of the three-class classifiers to discriminate between task-related samples (consisting of left and right attempted movement samples) and rest samples, as can be seen in supplementary material Fig. 10. We can see that when the task vs rest binary classification was above chance (see Fig. 3), the confusion matrices have a diagonal structure; in the other cases, the classifier shows a propension to recognize samples as belonging to the task class, likely because it is more represented in the balanced right vs left vs rest training set. In Fig. 6(b), we show that the three-class decoding produces above chance left vs right discrimination during three of the four sessions from patient P1.

## DISCUSSION

In the present work, we investigated the possibility to decode lower-limb movement attempts in four patients with different SCI severity, namely, one ASIA C (P1), one ASIA B (P4), and two ASIA A (P2-3) patients, across four experimental sessions. We visualized ERD/ERS patterns of motor attempts relative to rest (Fig. 1, supplementary material Figs. 3 and 4), noticing that the most notable components emerged as ERDs in lower frequency bands (theta, alpha, and beta). Statistical analyses confirmed significant spectral differences in central regions (Fig. 2, supplementary material Fig. 5), coherently with the literature on upper-limb movement-related EEG decoding^61,62^ and on hand and foot movement decoding.^36,51^

Single-trial classification of attempted movement vs rest generally exceeded the chance level (Fig. 3), aligning with the statistical differences observed in spectral features. The substantial decrease in patients P2 and P3 in accuracy of sessions two-four compared to the first may be caused by a difficulty to maintain motivation throughout lengthy open-loop calibrations where complete SCI patients have neither kinesthetic nor visual feedback. Performance may be increased through the use of closed-loop feedback^63^ or virtual reality training.^63–65^ However, as we were interested in establishing baseline feasibility rather than optimizing performance, we deliberatively employed an open-loop protocol to minimize potential confounds related to adaptive learning or feedback-based performance modulation. We note that all patients were enrolled in a clinical trial investigating the effects of epidural electrical stimulation throughout a three-month trial period and performed daily rehabilitation sessions, both with and without epidural electrical stimulation. Consequently, factors such as physical fatigue, emotional state, and the level of engagement during each experimental session may have influenced cortical activation and, in turn, decoding performance. At the same time, this variability mirrors real-world conditions, as patients in everyday life naturally experience fluctuations in fatigue, attention, and motivation due to their clinical condition. Therefore, our experimental setup approximates realistic usage scenarios. Future work should consider psycho-physiological influences more systematically, for example, by correlating decoding performance with daily fatigue or attention scores obtained from questionnaire assessments.

When we consider sessions less influenced by daily psychological factors, particularly the first session for each patient, our performances are in line with those presented for motor imagery and motor attempts in similar EEG-based studies, with accuracy generally ranging between 63% and 75% in complete SCI and 75%–85% in healthy subjects. Indeed, the trend toward higher decoding accuracy in the incomplete patient (P1) compared to the motor-complete patients (P2–P4) is consistent with previous studies, showing that incomplete SCI is associated with cortical activation and motor imagery performance more similar to healthy subjects,^66,67^ whereas complete injuries generally exhibit lower accuracies and altered ERD/ERS patterns.^68–70^ It is also important to note that most of these prior investigations focused on upper-limb movements, while this work examined lower-limb movement attempts, which typically produce weaker EEG signals due to the deeper and more medial cortical representations of leg areas. Therefore, future studies should include larger homogeneous cohorts to further validate our findings.

The choice of a machine learning classifier based on decision trees was primarily motivated by its robustness and reliability in scenarios involving limited and heterogeneous datasets.^71^ Methods such as XGBoost exhibit greater control of overfitting compared to deep neural networks, which typically require large and homogeneous datasets and extensive hyperparameter optimization to generalize effectively.^72–74^ In addition, prior studies have reported that classifier methods based on decision trees achieve competitive or superior results in EEG decoding tasks, including motor imagery,^75–77^ even when data availability is limited.

Results reported in Fig. 4 suggest that approximately 1 s of motor attempt signal is sufficient to identify reliably a movement attempt, allowing a balance between temporal resolution and classification accuracy. This time window is suitable for applications that require discrete or tonic control rather than continuous modulation. For instance, such signals could be used to trigger cyclic stimulation protocols to initiate functional actions, such as standing up or beginning to walk sequence, similar to the protocols employed in Refs. 14, 15, 21, and 78. These simplified control strategies may be more robust and feasible than instantaneous control in the presence of noisy or variable EEG signals. Future paradigms should, therefore, align the complexity of control tasks with reliability of the neural decoding approach, considering factors like fatigue and the need for real-time adjustments in a more intuitive and adaptive manner.

The robust discriminability between task and rest states, regardless of the specific executed movements (supplementary material Fig. 7), further opens the possibility for intuitive control systems. We might have expected attempted hip movements to be systematically harder to identify the knee movements due to the placement of their corresponding cortical areas, but our results indicate otherwise. This finding suggests the possibility of developing decoders that selectively control specific joint movements, allowing patients to control electrical stimulation by attempting the desired movement directly, without requiring substitution mechanisms (such as controlling knee extension through attempted hip flexion). In addition, in the case of patient P1, it was possible for three out of four sessions to perform above-chance three-class decoding (Fig. 6), which may allow to develop flows of control that require to discriminate between two-three classes at any time point, where the subject should operate high-level choices to modify the active electrical stimulation protocol.

Discrimination between left vs right movements and between hip vs knee movements was possible in some cases, though generally, it did not produce above-chance decoding accuracy (Fig. 5). In general, left-right discrimination achieved higher accuracy than hip-knee discrimination. This aligns with literature: for example, left vs right lower-limb movement decoding in healthy subjects produced only slightly above-chance (average 63%) accuracy levels,^79^ increasing to approximately 80% using post-imagery beta rebounds.^80^ However, such an approach is not viable for real-time intuitive control, as it would delay decoding until after movement intention has ended, introducing a temporal mismatch between intention and execution that could diminish rehabilitative benefits.^81^ It is expected that the performance on paralyzed subjects will be much lower. Indeed, in Ref. 64, some hint that the neural response may be lateralized has been provided using common spatial patterns, but left vs right decoding has not been explicitly attempted, relying on a state-machine to establish whether left or right steps needed to occur. In line with these observations, our analyses using CSP in the task vs rest paradigm also yielded generally lower classification accuracy compared to using power spectral features, suggesting that the spatial filtering introduced by CSP may not effectively capture the distributed cortical dynamics associated with these movements (supplementary material Fig. 11). Hip vs knee decoding showed, in general, chance-level performances, likely due to anatomical constraints. Cortical areas controlling proximal and distal lower movements are, indeed, located in close proximity,^82^ reducing their spatial separability in EEG signals. Additionally, the presence of complete lesions in most patients likely impaired their ability to selectively focus on evoking the intended movements. Invasive solutions like ECoG^30^ show a much higher accuracy in left vs right discrimination, coherently with the much higher spatial resolution resulting from having some sensors directly on the left and right hemispheres, thus allowing a neat separation between left and right cortical correlates. Still, the classification performance decreases substantially when trying to decode the movement of different joints, analogously to what we have shown here.

Multi-window classification analysis examining left vs right and hip vs knee showed that classification accuracy can improve by combining multiple predictions (Fig. 5). Although these accuracy gains are modest on average, they lead to an important improvement in the number of above-chance classifications, hinting at the presence of detectable neural modulations and suggesting that enhanced performance may be achieved with larger training datasets or virtual reality feedback.

A fundamental step toward clinical application of noninvasive decoding will consist of characterizing the available degrees of freedom to control a neuroprosthesis, such as through electrical stimulation or an exoskeleton. In this study, we acknowledge that decoding accuracy approached chance level in some sessions, even for the best-performing participants, reflecting the inherent difficulty of decoding lower-limb movements from scalp EEG signals. Nevertheless, our longitudinal design (collecting multiple sessions over several months of intensive rehabilitation with SCS) allowed us to assess the temporal evolution and stability of decoding performance, providing insights into how EEG-based discrimination may changeover time. These findings suggest that, although multi-class decoding remains challenging, simpler control strategies such as binary switches or activity-specific stimulation program selection based on state-machines^64^ may be realistically achievable using EEG recordings, without the need for more invasive approaches.

Overall, our results should be regarded as a baseline assessment of feasibility rather than as evidence of clinical-level performance. They demonstrate the potential of EEG to capture discriminative features related to lower-limb movements and to serve as a noninvasive input for triggering or modulating specific SCS programs. Future studies with larger cohorts of spinal cord injury patients will be essential to further explore the potential and limitations of EEG as a control signal for neuromodulation systems.

## METHODS

### Data acquisition

#### Patients

Four participants with chronic thoracic SCI were enrolled in this study. According to the American Spinal Cord Injury Association (ASIA) impairment scale,^83^ P1 was classified as ASIA C (motor and sensory incomplete), P2 and P3 were ASIA A (motor and sensory complete), and P4 was ASIA B (motor complete and sensory incomplete). Patients' demographic information and clinical features are summarized as follows:

• Patient P1 was a 33-year-old male patient with T11-T12 SCI, classified as ASIA C, and was enrolled in November 2023 in our trial. He had a history of SCI occurred 4 years earlier, resulting in a T12 vertebral fracture with T11-T12 myelopathy. Following the injury, he underwent an emergency T10-L2 fusion and decompression of the spinal canal. At the time of trial enrollment, he was classified as AIS C only due to the presence of traces of contraction in the proximal hip flexor and extensor muscles bilaterally (MRC grade 1); no voluntary muscle contractions (MRC grade 0) were observed in the other muscles of the lower limbs. The patient had severe central and peripheral damage almost complete from L4 to S1 and partial at the L2–L3 level.
• Patient P2 was a 27-year-old male with T7 SCI, classified as ASIA A (motor and sensory complete). He was enrolled in May 2024 in our trial. He had a history of SCI occurred 32 months earlier, resulting in a T7 and T8 vertebral fracture. Following the injury, he underwent an emergency T5–T10 fusion and decompression of the spinal canal.
• Patient P3 was a 33-year-old male with a T4 spinal cord injury (SCI), classified as ASIA A. He was enrolled in the trial in July 2024. His SCI occurred 36 months prior, resulting in a T4 vertebral fracture with associated myelopathy extending from T2 to T6. Following the injury, he underwent emergency spinal fusion from T2 to T6 and decompression of the spinal canal.
• Patient P4 was an 18-year-old male patient with T8 SCI, classified as ASIA B (motor complete and sensory incomplete). He was enrolled in September 2024 in our trial. He had a history of SCI occurring 12 months earlier, resulting in a T7 and T8 vertebral fracture. Following the injury, he underwent an emergency T4–T11 fusion and decompression of the spinal canal.

All enrolled patients suffered from lower-limbs neuropathic chronic pain (paresthesia and/or burning dysesthesia in both lower limbs), following a traumatic SCI at the thoracic level. They were able to independently transfer from a supine to a seated position and from a seated to a supine position with compensatory movements. They also had neurogenic bladder and bowel function, necessitating self-catheterization and bowel irrigation procedures. All patients signed an informed consent for the participation to these experiments.

#### Experimental protocol

**FIG. 7.**
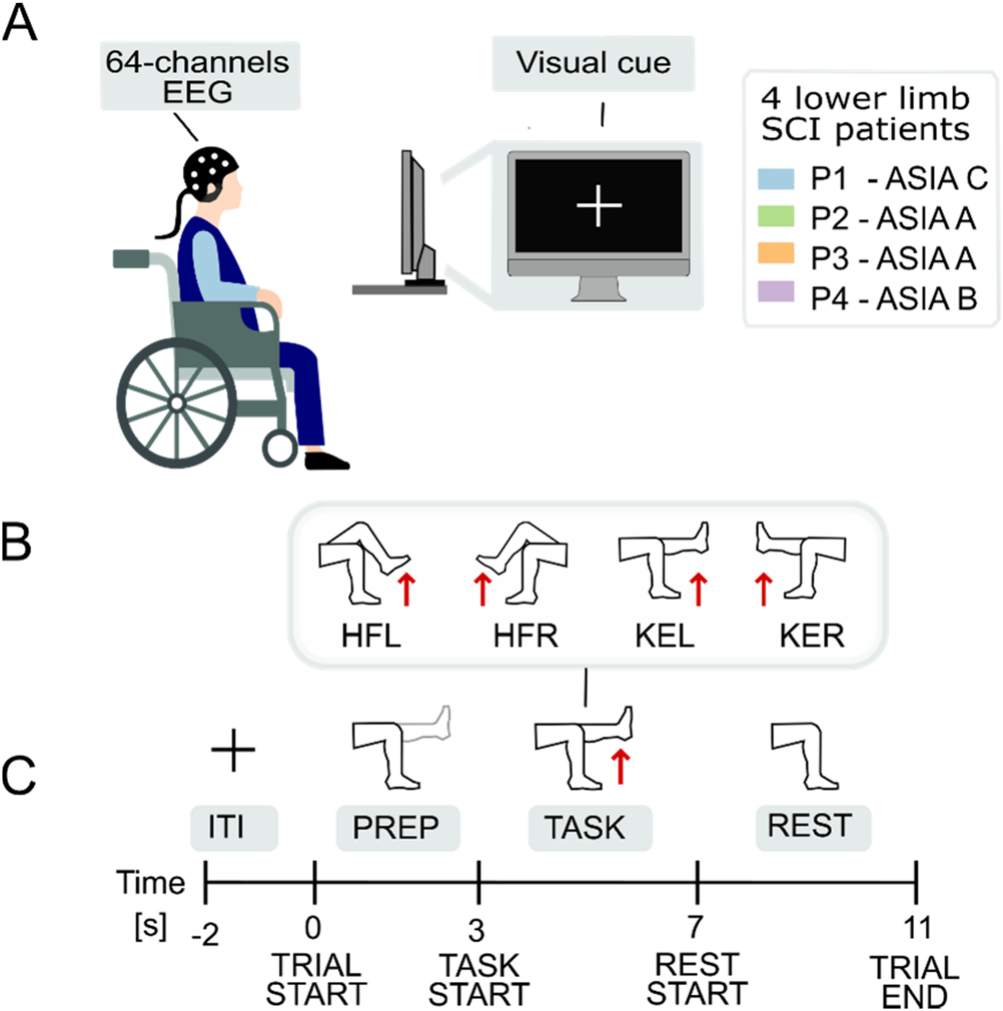
Experimental design. (a) Experimental setup: subjects were seated in their wheelchair facing a laptop screen, which displayed visual cues throughout the experiment. (b) Targeted lower-limb movements: left hip flexion (HFL), right hip flexion (HFR), left knee extension (KEL), and right knee extension (KER). (c) Trial structure: each trial included a preparation phase (PREP—motor preparation), an execution phase (TASK—attempted movement), and a rest phase (REST), with trials separated by a 2-s intertrial interval (ITI).

The experimental design has been already briefly described in Ref. 84 and is illustrated in Fig. 7. Subjects were seated in a wheelchair in front of a standard laptop screen, employed to provide visual cues on the tasks [Fig. 7(a)]. The visual presentation of the experiment was created using Psychtoolbox package in MATLAB. Each subject performed four experimental sessions, each consisting of 30 repetitions (trials) of each one of four movements, common for all patients: left hip flexion, right hip flexion, left knee extension, and right knee extension [Fig. 7(b)]. Movements were proposed in a different randomized order during each session. At the onset of a trial, a visual cue indicating the target movement for the trial was shown, followed by a 3 s preparation period where the patient was instructed to visualize the movement without attempting it (motor preparation task), a 4 s execution period where the patient was instructed to perform/attempt the target movement (maintaining “maximum contraction” throughout the period of time), and a static rest phase of 4 s duration. Trials were separated by an intertrial interval of 2 s duration [Fig. 7(c)]. The four experimental sessions were performed at two-week intervals for patient P1 and at four-week intervals for patients P2, P3, and P4. The unequal spacing of experimental sessions across patients was caused by clinical needs unrelated to the present study.

#### EEG acquisition

EEG data were recorded with a sampling frequency of 1 kHz using a 64-channels waveguard original ANT-Neuro cap (https://www.ant-neuro.com/products/waveguard-original) with sensors/channels positioned on the scalp according to the 10–20 International System.^85^ Channel CPz acted as reference electrode and channel AFz as ground electrode. EEG recording and synchronization with the visual cues were performed using Lab Streaming Layer (https://github.com/sccn/labstreaminglayer). After starting EEG recording, a trigger signal was sent at the start of the experimental presentation to denote the beginning of the experiment. Additional trigger signals were sent during the experiment to identify individual trials. Each one of the four movements was associated with a unique trigger signal, which was sent at the presentation of the corresponding visual cue.

### Data analysis

#### EEG preprocessing

After the acquisition, data were exported and analyzed using Python. Most EEG-specific processing was performed employing functionalities within the MNE toolbox for Python.^86^

Preprocessing consisted of EEG bad channel rejection, artifact rejection through independent component analysis (ICA), filtering, and epoching. We rejected channels with an impedance higher than 100 kΩ, for an average of two channels removed per session. Signals were then bandpass filtered in (1, 100) Hz, to include theta (4–8 Hz), alpha (8–12 Hz), beta (12–30 Hz), and gamma (30–100 Hz) bands. These four bands were chosen as they are those normally included in movement imagery/attempt decoding.^87–89^ Filtering was done using the mne.Raw.filter function to create a one-pass, zero-phase, non-causal FIR filter designed using a window method with Hamming window. We performed an ICA with 12 components to remove ocular artifacts, such as blinks and horizontal eye movement. ICA was implemented using the Picard algorithm within the mne.preprocessing.ICA method.^90^ To reduce the size of data, we resampled at 256 Hz with anti-aliasing filtering, so that we did not distort the underlying signal. Finally, we epoched the signal setting as time point zero the presentation of the movement preparation visual cue, and as the final time point the end of the rest period [Fig. 7(c)]. The resulting epochs were manually inspected, and the final dataset employed in this study consists of the epochs where severe movement or electrical communication artifacts were absent. Table I reports the final number of epochs acquired in each session for each patient and movement.

**TABLE I.** Number of employed trials per patient, session, and task.

Patient ID	P1 [HFL, HFR, KEL, KER]	P2 [HFL, HFR, KEL, KER]	P3 [HFL, HFR, KEL, KER]	P4 [HFL, HFR, KEL, KER]
1	[31, 29, 30, 30]	[27, 29, 25, 29]	[31, 31, 31, 32]	[22, 22, 24, 20]
2	[17, 14, 17, 13]	[29, 30, 29, 28]	[29, 29, 30, 30]	[29, 30, 29, 29]
3	[26, 29, 28, 26]	[19, 20, 19, 17]	[27, 25, 27, 24]	[29, 30, 27, 29]
4	[29, 26, 25, 28]	[26, 27, 28, 27]	[18, 20, 20, 20]	[28, 30, 30, 29]

#### Band-wise ERD/ERS analysis

We inspected topographic band-wise ERS/ERD averaged across trials to characterize the cortical correlates of attempted movement. We computed band-wise ERD/ERS as the relative value,

$$\text{ERD}/\text{ER}S_{\text{band}}=\frac{{\text{Pow}}_{\text{band}}^{\text{task}}-{\text{Pow}}_{\text{band}}^{\text{rest}}}{{\text{Pow}}_{\text{band}}^{\text{rest}}},\;\text{with}\;\text{band}=\theta ,\alpha ,\;\beta ,\gamma ,$$ (1)

where negative values indicate “desynchronization” of task with respect to rest, and positive values indicate “synchronization” of task with respect to rest.^91^ We considered task and rest periods inside a single trial, namely, the motor attempt period and the following rest period. We discarded the first second of both rest and task conditions to avoid any spurious component from sensory processing of the visual cues provided to subjects. Band-wise power was computed as the mean squared value of the EEG signal bandpass filtered according to each characteristic band. We excluded from the analysis channels on the outer scalp circumference (supplementary material Fig. 2), as they are, in general, more subject to movement-related artifacts.^92^

#### Statistical characterization of spectral differences

We performed statistical analysis to evaluate significant differences between movement and rest phases across the four frequency bands. We estimated the power spectral density (PSD) using the multitaper method in the 4–100 Hz frequency range.^93,94^ We focused only on the channels over the “central” region of the scalp, composed of channels C1, C2, C3, C4, C5, C6, CP1, CP2, CP3, CP4, CP5, and CP6, since they cover sensory-motor areas placed around the central sulcus of the brain.^95,96^ Epochs were segmented into movement (4–7 s) and rest (8–11 s) periods, and evoked PSDs were obtained by averaging across trials for each condition.

To assess condition-related differences, a non-parametric cluster-based permutation test (mne.stats.permutation_cluster_test) was applied on the PSD data [13]. Spatial adjacency between channels was computed using mne.channels.find_ch_adjacency, which accounted for channel layout in the clustering process. The permutation test was one-tailed (tail = 1), and clusters were considered significant at p < 0.05. For each of the 13 central channels, frequencies contributing to significant clusters were stored, allowing identification of frequency bands where movement-related spectral power differed significantly from rest.

### Classification analyses

#### Classification tasks

We performed several classification tasks. Depending on the specific case, we selected different time intervals taken from the trials, and we computed the PSD using the multitaper method between the 4 and 100 Hz frequency range. In selecting the interval of each dataset, the first second of each phase [preparation (0–1 s), movement execution (3–4 s) and rest (7–8 s)] was excluded to make sure we were not using data spurred by the presentation of the visual cue on the screen. As for the statistical characterization of spectral differences, we only included central EEG channels (C1, C2, C3, C4, C5, C6, CP1, CP2, CP3, CP4, CP5, and CP6) in the analysis. The PSD values from the selected intervals and channels were then used to build the dataset that was fed into the classifier. All classification tasks were performed separately for each subject and for each session.

We will now list all classification tasks that were performed and indicate the time intervals that were used to form the relative dataset:

(1) *All movement attempts vs rest classification*: We used all available data for each session, irrespective of the target movement. The two classes were formed by 3-s windows during movement execution (4–7 s) and rest phases (8–11 s) [supplementary material Fig. 1(a)]. To evaluate which feature representation yielded the best classification performance, the same decoding analysis was conducted using either (i) PSDs computed from central channels, (ii) PSDs computed from all available channels, or features extracted via a common spatial patterns (CSP) decomposition implemented with mne.decoding.CSP, using (iii) 5 or (iv) 15 components.
(2) *Single movement attempt vs rest classification*: We studied if there was a preference of distal vs proximal muscles or right vs left side in the movement attempt vs rest classification. To do so, we performed movement execution (4–7 s) vs rest (8–11 s) classification for each movement separately [supplementary material Fig. 1(a)].
(3) *Minimal trial length to perform movement attempt vs rest classification*: We studied how much data in terms of trial duration are needed to obtain statistically above-chance results in movement attempt vs rest decoding. We tested intervals of 0.05, 0.1, 0.2, 0.5, 1, 2, and 3 s. Time intervals were taken starting 1 s after the beginning of movement/rest phases to form the two classes [supplementary material Fig. 1(b)].
(4) *Movement preparation vs rest classification*: Performed on all available data for each session, irrespective of the target movement. We considered 2-s windows during the preparation (1–3 s) and rest phases (9–11 s) [supplementary material Fig. 1(c)].
(5) *Single-trial classification of task vs task*: We performed left vs right movement and hip vs knee movements, considering (4–7 s) of each movement [supplementary material Fig. 1(d)].
(6) *Three classes decoder:* We performed a three classes decoder to classify right vs left vs rest, considering 3-s windows during movement execution (4–7 s) and rest phases (8–11 s) [supplementary material Fig. 1(e)].
(7) *Multi-window classification of task vs task decoding:* We performed both left vs right movement and hip vs knee movements multi-window classification [supplementary material Fig. 1(f)]. We started by taking 3-s windows of each movement (4–7 s). Then, for each epoch, we segmented the 3-s movement interval into overlapping windows of 1.0 s duration, using a sliding step of 0.1 s. For each window, we computed the power spectral density (PSD) within the 4–100 Hz frequency range using the multitaper method. Predicted epoch labels were then determined by taking the statistical mode of the predicted labels from all windowed samples within the epoch. In this way, the number of test labels is still equal to the number of test epochs, but we leverage multiple predictions from shorter segments within each epoch to increase performance.

#### Employed machine learning

For each patient and session, we implemented XGBoost (eXtreme Gradient Boosting) classifier using the sklearn function xgboost.XGBClassifier. We employed a Monte Carlo resampling approach^97^ with 50 iterations: For each iteration, we shuffled the dataset and randomly extracted a predefined number of test trials (30 trials) while using the remaining dataset to train the XGBoost model. Training and test sets were balanced in order to have the same number of samples per task in each set.

#### Chance-level determination and comparison with decoding performance

To determine whether our results were above chance, we computed random baseline scores for each classification task. Random baseline scores represent the expected performance of a model making uninformed guesses and were compared to our decoding performance.

For binary classification tasks, random predictions were generated by sampling from a uniform distribution over the interval (0, 1) and applying a threshold of 0.5 to simulate random guessing across classes. For the three-class classification baseline, random predictions were generated by sampling uniformly from the set of class labels (0, 1, 2). To determine whether the classifier significantly outperformed the random baseline, we conducted a Wilcoxon signed-rank test (scipy.stats.wilcoxon) for each subject and session. To correct for multiple comparisons, we applied a Bonferroni correction, adjusting each p-value by 16 (number of subjects × number of sessions).

## SUPPLEMENTARY MATERIAL

See the supplementary material for Figs. 1–11, which provide additional details on dataset labeling strategies, sensor layout, ERD/ERS topographies, spectral power contrasts between task and rest, and extended classification performance analyses for multiple decoding tasks (preparation vs rest, right vs left, hip vs knee, and multi-class decoding).

## Data Availability

The data that support the findings of this study are available from the corresponding author upon reasonable request.
